# Sar Ship Detection Based on Convnext with Multi-Pooling Channel Attention and Feature Intensification Pyramid Network

**DOI:** 10.3390/s23177641

**Published:** 2023-09-03

**Authors:** Fanming Wei, Xiao Wang

**Affiliations:** College of Computer and Information Engineering, Nanjing Tech University, Nanjing 211816, China; 202161220083@njtech.edu.cn

**Keywords:** Convnext, feature intensification pyramid network (FIPN), SAR ship detection, multi-pooling channel attention (MPCA), top-level feature intensification (TLFI) module

## Abstract

The advancements in ship detection technology using convolutional neural networks (CNNs) regarding synthetic aperture radar (SAR) images have been significant. Yet, there are still some limitations in the existing detection algorithms. First, the backbones cannot generate high-quality multiscale feature maps. Second, there is a lack of suitable attention mechanisms to suppress false alarms. Third, the current feature intensification algorithms are unable to effectively enhance the shallow feature’s semantic information, which hinders the detection of small ships. Fourth, top-level feature maps have rich semantic information; however, as a result of the reduction of channels, the semantic information is weakened. These four problems lead to poor performance in SAR ship detection and recognition. To address the mentioned issues, we put forward a new approach that has the following characteristics. First, we use Convnext as the backbone to generate high-quality multiscale feature maps. Second, to suppress false alarms, the multi-pooling channel attention (MPCA) is designed to generate a corresponding weight for each channel, suppressing redundant feature maps, and further optimizing the feature maps generated by Convnext. Third, a feature intensification pyramid network (FIPN) is specifically designed to intensify the feature maps, especially the shallow feature maps. Fourth, a top-level feature intensification (TLFI) is also proposed to compensate for semantic information loss within the top-level feature maps by utilizing semantic information from different spaces. The experimental dataset employed is the SAR Ship Detection Dataset (SSDD), and the experimental findings display that our approach exhibits superiority compared to other advanced approaches. The overall Average Precision (AP) reaches up to 95.6% on the SSDD, which improves the accuracy by at least 1.7% compared to the current excellent methods.

## 1. Introduction

SAR is an active Earth observation device that can be employed on various platforms including space stations, aircraft, spacecraft, and other equipment [1].

It offers a range of advantages, including ground penetration, all-weather capability, and continuous operation, making it widely adopted by many countries. SAR finds applications in various fields such as route planning, ocean detection, natural disaster warning, and military operations, demonstrating its high value for civil, military, and commercial purposes [2,3]. Marine detection, as a significant domain of SAR system applications, has gained increasing attention from researchers due to its practical value [4].

Currently, detecting ships in SAR images has emerged as a crucial technique in marine detection, garnering significant attention from researchers. SAR ship detection has extensive applications. In the military, detecting the location of specific targets can aid in tactical planning and enhance maritime defense warning capabilities. In civilian use, detecting smuggling and illegal fishing vessels can assist in monitoring and managing maritime transportation. With the rapid advancements in SAR technology, including the deployment of airborne and spaceborne radars, there has been a substantial increase in the acquisition of SAR image data. Consequently, the timely and efficient extraction of target information from vast SAR image datasets has become paramount.

The traditional SAR ship target detection approach mainly adopts a constant false-alarm rate (CFAR) [5], which first assumes that ocean clutter obeys certain distribution, and then artificially sets false alarms according to the difference between ship and clutter gray level, so as to detect ship targets. But there are many problems with this approach. First, the CFAR algorithm judges the target pixel in accordance with a pixel value of a single intensity, and the judgment basis is insufficient, resulting in many false alarms. Second, because the gray value of the target is in a specific range, the detection result is prone to fracture. Third, this method needs to set many windows, which also greatly increases the amount of computation. Fourth, this method cannot meet the requirements of high-resolution SAR images. Therefore, a convenient and accurate method is urgently needed to realize the detection task.

With the application and popularization of deep learning, target detection in optical images has made remarkable headway. The target detector is mainly partitioned into two kinds. One refers to the one-stage detector, while the other corresponds to the two-stage detector. One-stage detectors mainly include OverFeat [6], SSD [7], Retinanet [8], EfficientDet [9], YOLOV1-V7 [10,11,12,13,14,15,16], TOOD [17], etc. The feature of the one-stage detector is to directly regress the position of the target and category through the detector, which is different from the detection mechanism of the two-stage detector. The two-stage detector first puts forward some proposals to obtain the approximate position and prospect probability of the target frame through the network of the first stage, and the second stage is to regress the corresponding location and category of the target frame through another network. Typical two-stage detectors contain Faster R-CNN [18], Fast R-CNN [19], SPPNet [20], R-CNN [21], R-FCN [22], Cascade R-CNN [23], Dynamic R-CNN [24], etc. Taking inspiration from the achievements in target detection and recognition in the area of optical images, the target detector in the optical area is gradually being applied to SAR ship images, which also brings new opportunities for SAR ship target detection. At the moment, the success of deep learning in the field of natural language processing has also given us some insights into the field of semantic information enhancement research [25].

Up to now, an increasing number of researchers improved deep learning methods for achieving SAR ship detection tasks. Li [26] put forward a method based on Resnet50 to improve the detection accuracy for SAR ships. Hu [27] et al. introduced deformable convolution to construct an attention module to better obtain the ship characteristics. Li [28] et al. put forward a feature pyramid network to learn hierarchical spatial features, thereby addressing the problem of multi-scale detection. Zhou [29] put forward a pyramid network with transferable features, which can effectively solve the issue of difficult detection of dense multi-scale targets. Ke [30] put forward a SAR ship detector using a feature enhancement feature pyramid network (FEFPN) to enrich the semantic information of feature maps and using Swin Transformer as the backbone to improve multiscale feature maps. Gao [31] balanced precision and speed by introducing a threshold attention module. To enhance the accuracy of small target detection, Zhou [32] put forward the low-level path aggregation network to find a suitable single scale for detection. To achieve strong generalization and eliminate the reliance on knowledge adaptation, Zhang [33] proposed an optimal detector through a self-training object detection approach using automatic deep learning.

However, the following problems still exist in SAR ship image target detection and recognition. First, although Ke proposed the FEFPN to improve the semantic information of shallow feature maps, the semantic information is not rich. In addition, the semantic information quality of the top-level feature maps is originally affluent, but after the channel reduction, the semantic information quality is weakened. Third, although Lin put forward a squeeze and excitation module to suppress false alarms, the module only uses one pooling method which may lose many SAR ship features. Fourth, although Ke thinks that a transformer can establish remote dependencies for SAR ship feature extraction, we believe that a pure convolutional network still has some advantages for SAR ship detection.

Our contributions mainly include the following four points:

- We employ Convnext [34] as the backbone, and the feature extraction structure is a pure convolution extraction structure, the structure integrates the advantages of a convolutional neural network and attention mechanism extraction network, uses the existing convolutional structure, and adjusts relevant parameters to imitate the Swin Transformer [35] network. We can find the advantages of the network in the subsequent ablation experiments.
- For enhancing the semantic information of shallow feature maps, the FIPN module is designed. It uses higher feature maps for enhancing the semantic information in shallow feature mappings and uses TLFI for reducing information loss of top-level feature maps after channel reduction.
- We design an MPCA module that reassigns a weight to each channel, highlighting the important channels, and diminishing the useless channels. This module is assigned to the back of Convnext.
- We put forward a TLFI module. Because the top-level feature map has very abundant semantic information, however, after the channel reduction (768->256), the high-quality semantic information is weakened, in order to minimize this adverse effect, we believe that the loss of semantic information can be alleviated by using semantic information from different spaces.

In this paper, the Methodology section introduces the total method we proposed as well as the details of Convnext, FIPN, MPCA, and TLFI. The Experiments section mainly introduces two experiments, namely ablation experiments and comparative experiments. The datasets used in these two experiments are SSDD [36]. The Conclusion section summarizes this article.

## 2. Methodology

### 2.1. Proposed Method

**Figure 1:**
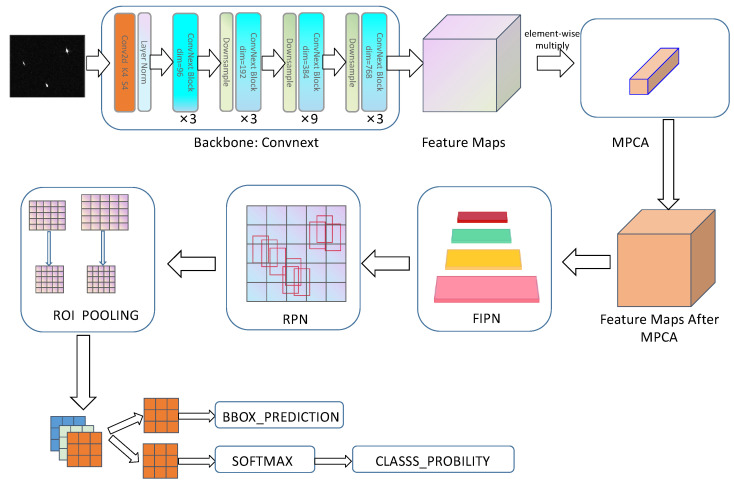
The structure of our method.

We propose a SAR ship detector based on Convnext with an MPCA module and FIPN. A two-stage detector is the primary structure of our proposed method. This two-stage detector is selected as the primary structure due to its benefits of affordability and high precision in detection. Our method is displayed in Figure 1, which has six parts, as follows: a backbone for feature extraction, an MPCA module for suppressing false alarms, a FIPN module for semantic information enhancement, a region proposal network (RPN) rather than the select search approach, an ROI pooling algorithm for unifying candidate region sizes, and, finally, two branches for category and regression. The detection process is as follows: the input image passes through Convnext to obtain four layers of feature maps. Their channel is 96, 192, 384, and 768, respectively. After that, the MPCA module reassigns a weight to every individual feature map to reduce the impact of redundant feature maps. FIPN performs semantic information intensification operation after the process of MPCA, to enhance the quality of semantic information in each layer of the feature map. Next, these feature maps are sent to RPN for the generation of proposals. The sizes of the proposals are unified after ROI POOLING, and then the proposals that are unified are sent to the following two branches for obtaining category and location information. The RPN, two branches, ROI, and corresponding structures in Faster R-CNN are consistent.

### 2.2. Convnext

The backbone shown in Figure 1 is the overall architecture of Convnext. The process is as follows: A SAR ship image has three channels, with a size of w × h, which can be represented as 3 × w × h. After processing in the first layer of Convnext, the number of channels and size remain unchanged. After the second layer of processing, the number of channels and size became 96 × ½ w × ½ h. After the third layer of processing, the number of channels and size became 192 × ¼ w × ¼ h. After the fourth layer of processing, the number of channels and size became 384 × ⅛ w × ⅛ h. After the fourth layer of processing, the number of channels and size became 768 × ⅟₁₆ w × ⅟₁₆ h. Convnext is a pure convolutional network, and all the structures in this model have been adopted in different neural networks. However, this does not prevent us from using a pure convolutional network for SAR ship detection. On the contrary, we believe that Convnext, based on the typical neural network and improved by imitating the structure of Swin Transformer, can combine the advantages of both pure convolutional networks and attention networks. At the same time, we also believe that pure convolutional networks ensure efficiency and simplicity, making them more suitable for SAR ship target detection.

**Figure 2:**
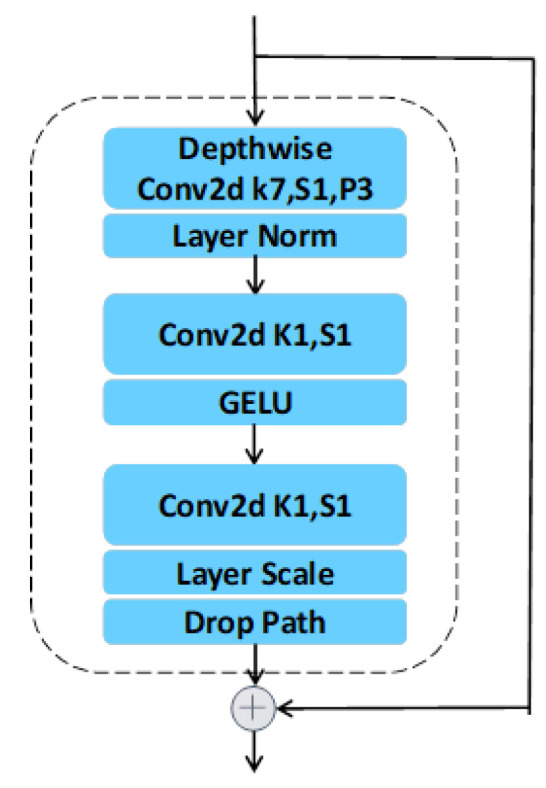
The structure of ConvnextBlock.

As demonstrated in Figure 2, each ConvnextBlock contains three parts. The first part has two processing structures. The first processing structure is a convolutional layer of size 7 and the stride of 1 and the padding of 3 are set. The second processing structure is a layer norm. In the second part, a convolutional layer using the convolution kernel size of 1 as well as a stride of 1 is first added, and then the GELU function is added for activation. The third part contains three structures, the first is a convolutional layer with size 1 and a stride of 1, followed by layer scale, and, finally, processed by the drop path.

**Figure 3:**
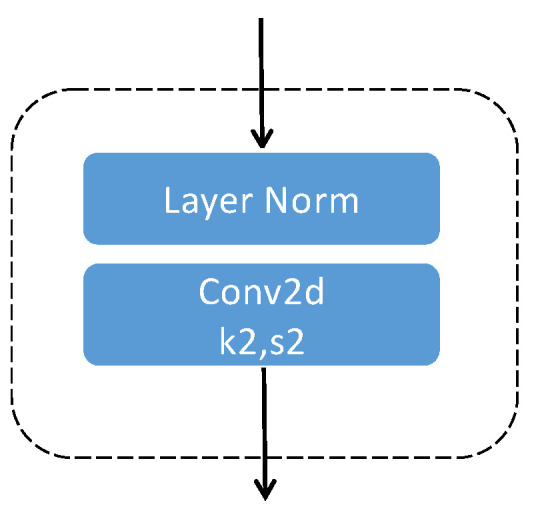
The structure of down-sampling.

As Figure 3 shows, each downsample contains two parts. The first part is layer normalization. The second part includes a convolutional structure with a kernel size of 2 as well as a stride of 2.

### 2.3. FIPN

**Figure 4:**
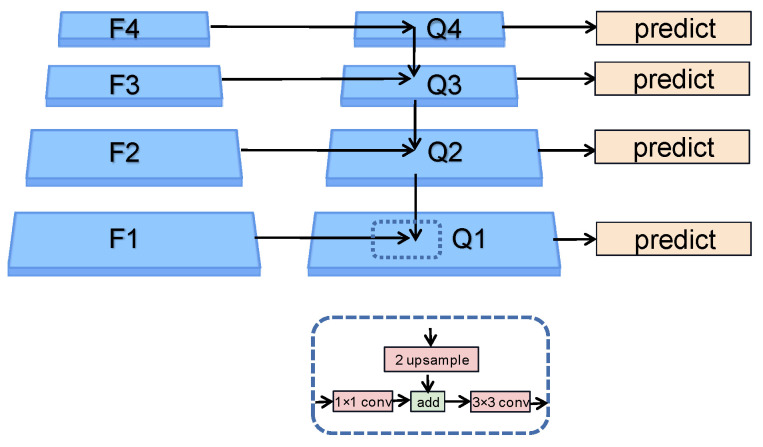
The structure of FPN.

For a better understanding of the advantages of FIPN, we compare FIPN with FPN [37]. The processing flow chart of FPN is demonstrated in Figure 4. FPN builds the feature pyramid structure from top to bottom after getting the feature maps (F1, F2, F3, F4) processed by Convnext. The number of channels and size for four feature maps is (96 × ½w × ½h, 192 × ¼w × ¼h, 384 × ⅟₈w × ⅛h, 768 × ⅟₁₆w × ⅟₁₆h). Since the channels of the four layers of the feature maps processed by Convnext are inconsistent (96, 192, 384, 768), each layer of the feature maps needs to go through a 1 × 1 convolutional structure to change the count of channels to 256. First, Fi (i starts from 4) is expanded by 2 times the original size through the nearest interpolation method, and then the corresponding elements are added to the F(i-1). In order to solve the problem of distortion of the original feature map caused by the superposition of the upsampling feature map and the original feature map, FPN uses a 3 × 3 convolution structure to remove confusion. After the above processing, Q4, Q3, Q2, and Q1 are obtained, and the semantic information in each layer feature maps can be expressed as four Equations (1–4).

Although FPN enriches the semantic information of shallow feature maps, FPN still has some problems. First, there is still much potential for improvement in shallow feature maps. Second, as a result of the reduction of the top layer feature maps channel from 768 to 256, the semantic information is lost, which leads to top-level feature maps with high-quality semantic information that cannot play their role. To solve the mentioned issues, we propose a feature intensification pyramid network (FIPN).

(1) Q4=F4

(2) Q3=F4+F3

(3) Q2=F4+F3+F2

(4) Q1=F4+F3+F2+F1

where Fi represents the semantic information of the i-th layer obtained through the feature extraction layer, and Qi represents the semantic information of the i-th layer feature map after FPN processing.

**Figure 5:**
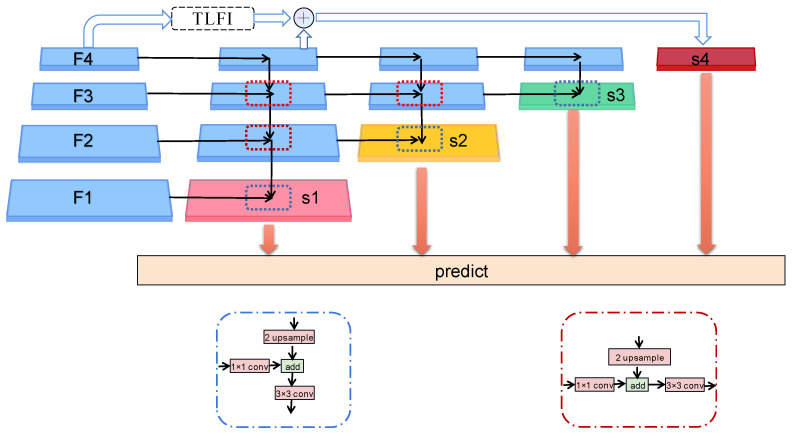
FIPN processing diagram.

As demonstrated in Figure 5, FIPN mainly contains four layers of feature maps, namely S1, S2, S3, and S4. Among them, S1 serves as the bottom layer of FIPN, S2 serves as the second layer of FIPN, S3 serves as the third layer of FIPN, and, finally, S4 serves as the top layer of FIPN. The FIPN algorithm is divided into four steps. First, the generation of S1 is consistent with the method of FPN. Then, S2 performs an algorithm operation from top to bottom based on the last three layers of features generated in the first step. Next, S3 is obtained by performing an FPN operation based on the last two layers of features generated in the second step. Finally, S4 is obtained by processing F4 through TLFI and adding it to the top-level feature map generated in the first step. After FIPN processing, S1, S2, S3, and S4 are obtained, and the information contained in each layer is shown in (5), (6), (7), and (8). The number of channels and size for four feature maps is (256 × ½w × ½h, 256 × ¼w × ¼h, 256 × ⅛w × ⅛h, 256 × ⅟₁₆w × ⅟₁₆h). Obviously, the feature maps processed by FIPN contain richer semantic information than FPN. It is important to mention that the top-level feature mapping after feature intensification is not used for the intensification process of shallow feature map semantic information. This is because the semantic information gap between the top-level feature maps is enhanced and the shallow feature maps can be large, and simple addition can cause great semantic confusion.

(5) S4=T(F4)

(6) S3=3F4+F3

(7) S2=3F4+2F3+F2

(8) S1=F4+F3+F2+F1

where *T* represents the effectiveness of TLFI, S4 represents the semantic information of the fourth layer feature map obtained after FIPN processing, S3 represents the semantic information of the third layer feature map obtained after FIPN processing, S2 represents the semantic information of the second layer feature map obtained after FIPN processing, and S1 represents the semantic information of the first layer feature map obtained after FIPN processing.

### 2.4. MPCA

**Figure 6:**
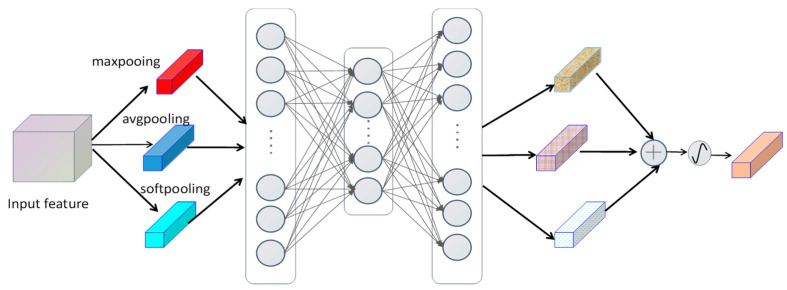
MPCA processing diagram.

**Figure 7:**
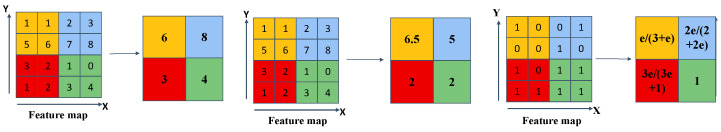
The schematic maps of three pooling methods. From left to right: max pooling, average pooling, and soft pooling.

To suppress false alarms, we propose the MPCA module, as demonstrated in Figure 6. This module mainly contains 3 components. The first component is to pool the feature maps through three pooling methods. They are maximum pooling, average pooling, and soft pooling. Maximum pooling can be easily implemented, and the maximum value in a feature map is extracted. Average pooling is getting the average of all values in the feature map. Soft pooling introduces the softmax function, which allocates the corresponding softmax weight per value in the feature map and adds all the values together. The schematic diagram of the three pooling methods can be shown in Figure 7. The maximum pooling, average pooling, and soft pooling formulas are (9), (10), (11), and (12), respectively. As for why we choose these three pooling methods, we will give the reasons here: First, during the soft pooling process, a softmax weight is added to each pixel and all are summed up, which can minimize the loss of information. Secondly, maximum pooling has a strong ability to remove noise, which is very effective for SAR images with high noise and complex backgrounds. Furthermore, average pooling can retain important feature information as much as possible. We believe that SAR images themselves have the features of high noise as well as complex backgrounds. Combining the advantages of these three pooling methods is very effective for target detection in SAR images. The second part mainly contains three dense layers, the quantity of nodes in the first layer is the quantity of input feature map channels, to save computing costs, the quantity of nodes in the second layer is reduced by 7 times compared with the first layer, and the quantity of nodes in the third layer matches the number of nodes in the first layer.

After passing through three dense layers, three different pooling vectors are generated. The third part is mainly obtaining a new vector by adding three vectors together. Finally, the vector is activated by the sigmoid.

(9) $$y=\underset{(i,j)\in R}{\max}x_{ij}$$

where *R* represents the coordinate set of the pooling kernel coverage area, $x_{ij}$ represents any feature value in *R*, and y represents the maximum pooling output.

(10) $$y=\frac{1}{\left|R\right|}\sum_{(i,j)\in R}x_{ij}$$

where *R* represents the coordinate set of the pooling kernel coverage area, |*R*| represents the number of feature values in *R*, represents any feature value in *R*, and *y* represents the average pooling output.

(11) $$W_{ij}=\frac{e^{x_{ij}}}{\sum_{(p,q)\in R}e^{x_{pq}}}$$

(12) $$y=\sum_{(i,j)\in R}w_{ij}\times x_{ij}$$

where *R* represents the coordinate set of the pooling kernel coverage area, $W_{ij}$ represents the weight of feature value in *R*, $x_{ij}$ represents any feature value in *R*, and *y* represents soft pooling output.

### 2.5. TLFI

**Figure 8:**
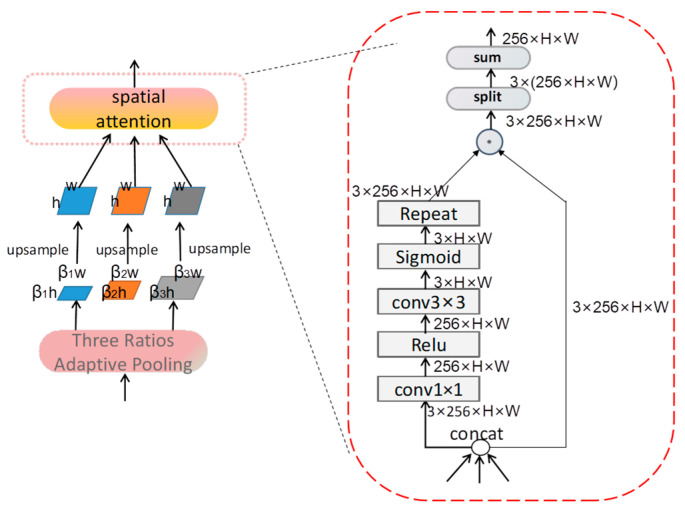
The structure of TLFI.

To solve the issue of semantic information loss in the top-level feature maps, we propose a TLFI module to solve the issue. The core idea of TLFI is to use three different spatial semantic information to make up for the loss of top-level semantic information. As demonstrated in Figure 8, first of all, F4 (w × h) is reduced by three proportions respectively, and the reduction proportions are selected as β1 = 0.1, β2 = 0.2, and β3 = 0.3. Then, we unify the three sizes of feature maps to the size of F4 using an upsampling method. The upsampling method is selected as the nearest interpolation method. To remove the adverse effects caused by the nearest interpolation method, we propose spatial attention to solve this problem. First, we connect the feature maps from three spaces; thus, the number of channels is increased from the original 256 to 768. Next, the channel is changed to 256 by a 1×1 convolutional structure, and for enhancing the sparsity of the feature maps, the Relu function is added after the 1×1 convolution structure. Then, we use the 3×3 convolutional layer to change the number of channels to 3, then three feature maps are nonlinear activated by using the sigmoid function, and the obtained three feature maps represent the weight maps of three different spatial feature maps. Finally, the spatial attention repeats three weight feature maps and multiplies the feature maps from three spaces with the corresponding weight maps to obtain three spatial feature maps after removing the confusion. Then, we sum up three spatial feature maps, and we believe that the resulting feature maps integrate the semantic information from three spaces.

## 3. Experiments

### 3.1. Experimental Dataset

**Figure 9:**
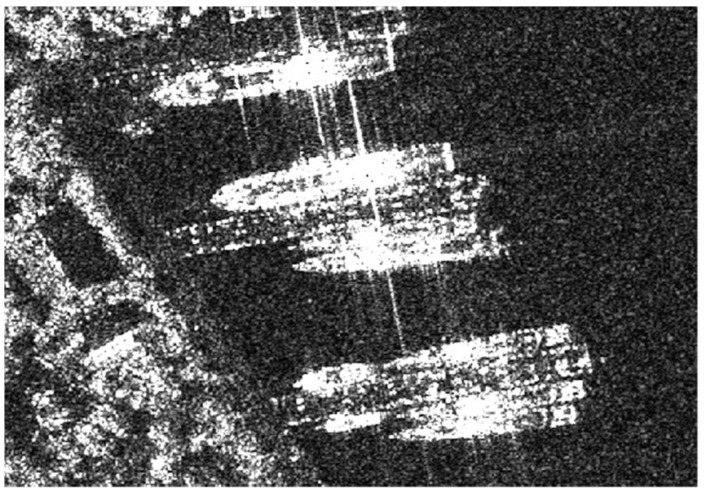
The image of inshore ship.

**Figure 10:**
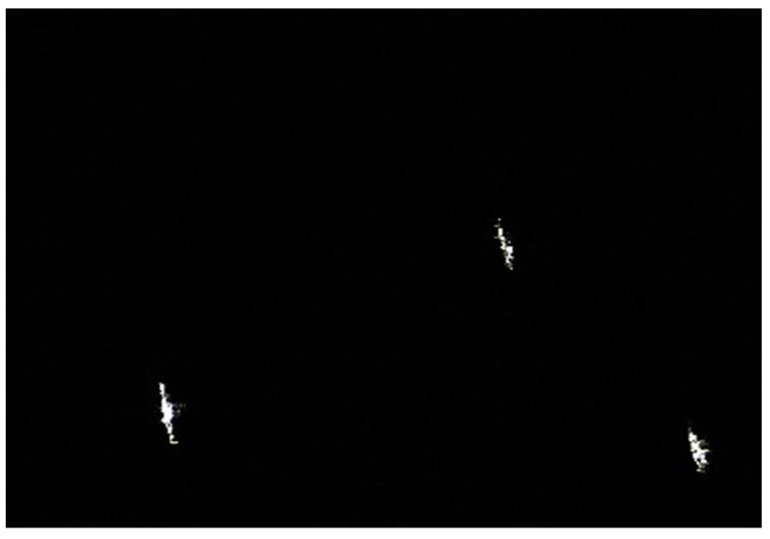
The image of offshore ship.

SSDD is the dataset we use for experiments. The dataset is specifically designed for SAR ship detection and has been broadly employed by many researchers. The ships in the images are very representative. The dataset contains 1160 images and 2456 ships. In this SAR dataset, the ships are mainly divided into inshore ships and offshore ships. Two images are displayed in Figure 9 and Figure 10.

### 3.2. Training Details

We allocate 80% of the dataset to the training set and 20% to the test set. The optimizer is set to Adam with an initial learning rate of 0.0001 and a weight decay of 0.05. Our experiments are conducted on a computer running Windows 10 with 32 GB of memory and an Nvidia GTX 1050 TI graphics card with 4 GB of memory. In our experiments, under the condition of maintaining the aspect ratio, image_scale is set to (608, 608) and the dataset is augmented using random flipping with a flip ratio of 0.5. The epoch is set to 20 since the model has been pre-trained, and appropriate results can be achieved by fine-tuning. We do not need too many iterations. All other configurations are consistent with the mMdetection toolbox [38]. Due to the limitation of graphics card memory, we configured the batch size to be 1 in our experiments.

### 3.3. Evaluating Indicator

In order to evaluate detectors, we select Average Precision (AP) and FPS as evaluation indicators. The AP is defined in (13), which is obtained by the recall in (14) and the precision in (15). FPS represents the number of predicted images per second. In our experiments, the condition of correct prediction is that the Intersection over Union (IoU) between the predicted bounding box and its ground truth is greater than 0.5.

(13) $$\mathrm{AP}=\int_{0}^{1}P(R)dR$$

(14) $$\mathrm{Recall}=\frac{\mathrm{TP}}{\mathrm{TP}+\mathrm{FN}}$$

(15) $$\mathrm{Precision}\;=\frac{\mathrm{TP}}{\mathrm{TP}+\mathrm{FP}}$$

where TP is an abbreviation for true positive, representing the number of accurately detected ships. FP is an abbreviation for “false positive,” representing the number of false alarms for ship targets. FN is an abbreviation for false negative, representing the number of missed ship targets.

### 3.4. Ablation Experiments

Here, we first carry out ablation experiments on Convnext and FIPN. To ensure the credibility of the experiment, we selected some advanced networks to compare with our method. As is well known, Resnet50 [39] is a convolutional neural network broadly employed within the area of SAR image detection, so we choose it as a control. Swin Transformer is a popular transformer network architecture. Its research shows that its performance is greatly improved compared to CNNs. Here, we choose it to compare with Convnext, which can better reflect the performance advantages of Convnext within the area of SAR ship detection. Focalnet [40] represents a new attention mechanism network with novel design ideas. To ensure fairness in the experiments, both Swin Transformer and Focalnet are implemented as small versions like Convnext. It is important to mention that except for the backbone, the other parts and parameters remain the same. From the data in Table 1, we can see that the detector using Resnet50 can achieve 93.2%, the detector using Focalnet can achieve 91.9%, the detector using Swin Transformer can achieve 93.9%, and the detector using Convnext can achieve 94.5%. Obviously, Convnext has the best performance. It can also be seen that pure convolutional networks still have considerable advantages over attention mechanism networks within the realm of SAR ship detection. As for ablations experiments about FIPN, we also chose some typical structures to serve as control groups. FPN is a widely used feature intensification method. PAFPN [41] is a new feature intensification method, and its fusion method is greatly improved compared to FPN. FPN_CARAFE [42] is a feature pyramid network using the carafe method. NASFPN is a new feature pyramid network based on neural architecture search. Except for the differences in the neck, the rest of the networks are uniform. As shown in Table 1, the detector with FPN can reach 94.5%, the AP of the detector with PAFPN can reach 92.5%, the AP of the detector with NASFPN [43] can reach 93.3%, the AP of the detector with FEFPN can reach 93.2%, the AP of the detector with FPN_CARAFE can reach 94.5%, however, and the AP of the detector with FIPN can reach 95.4%. Clearly, the AP of the detector with FIPN is the best, at least 0.9% higher.

Ablation experiments are then conducted to verify the effectiveness of TLFI. We compared detectors that do not use TLFI and using TLFI. Of course, except for TLFI, everything else is consistent. The experimental data is proven in Table 2. It is obvious that the AP of the detector using TLFI has reached 95.4%, the AP of the detector without TLFI is only 95.1%, and the AP is improved by 0.3%. Table 2 displays the results.

Next, we choose two advanced attention mechanism algorithms, such as squeeze-and-excitation (SE) [44] block and convolutional block attention module (CBAM) [45], which are compared with MPCA, as shown in Table 3. Except for the difference in attention mechanism algorithms, other structures are consistent. We select Convnext as the backbone and FIPN as the neck. We can see that after inserting SE, the AP is 95.3%. After inserting CBAM, the AP is 94.5%. After inserting MPCA, the AP reaches 95.6%, and it is clear that the AP of MPCA is the highest. As for why the AP drops by 0.1% after adding SE, we think that SE uses only one pooling method in the step of pooling the feature maps, and a single pooling method may omit important values. After inserting CBAM, the AP decreases sharply, with the AP down 0.9%, we think that CBAM uses spatial attention and channel attention, and channel attention may extract most of the ship’s features, but the use of spatial attention can weaken the correlation of ship features. Compared to SE and CBAM, the AP is improved by 0.3% and 1.1%, respectively.

### 3.5. Comparative Experiments

**Figure 11:**
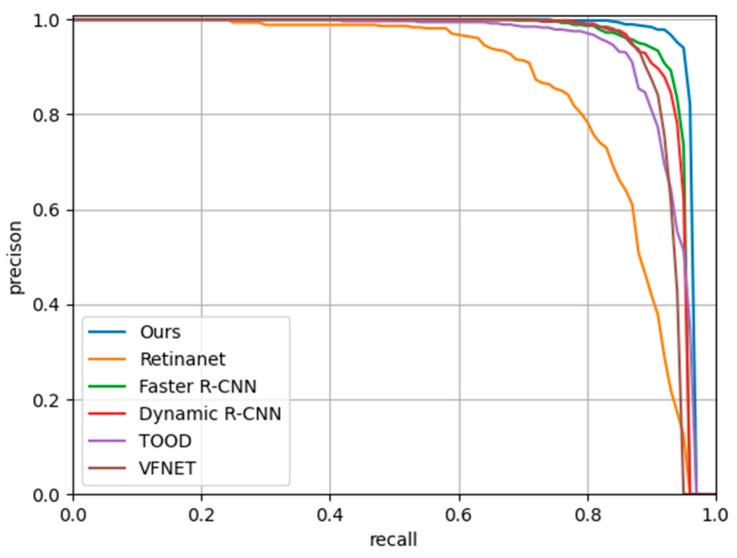
The precision–recall curves of four detectors on SSDD.

Following, five well-representative detectors are used to conduct the comparative experiments with our method. In these five detectors, the backbone and neck used are Swin Transformer and FPN. Since both SE and CBAM cause performance degradation, we do not use these two attention mechanisms in these five detectors. According to the data in Table 4, Faster R-CNN has an AP of 93.9%, Retinanet has an AP of 85%, Dynamic R-CNN has an AP of 93.7%, VFNet has an AP of 92.2%, TOOD has an AP of 92.3%; however, our method has reached 95.6%. Retinanet exhibits the poorest performance among all detectors. Within the area of SAR ship detection, we believe that one-stage algorithms, which directly obtain position and class information without a candidate proposal stage, may not be conducive to the accuracy of object detection. Because complicated background and noise disturbance in SAR images pose challenges, this approach is potentially unfavorable for precise target detection. Obviously, our method is the most advantageous compared to other methods, and the AP has increased by at least 1.7%. As shown in Figure 11, we make PR curves for four detectors. It is manifest that the area under the curve of the proposed approach is the largest, and our method has the highest precision under the condition of keeping the same recall.

### 3.6. Visualized Conclusions

**Figure 12:**
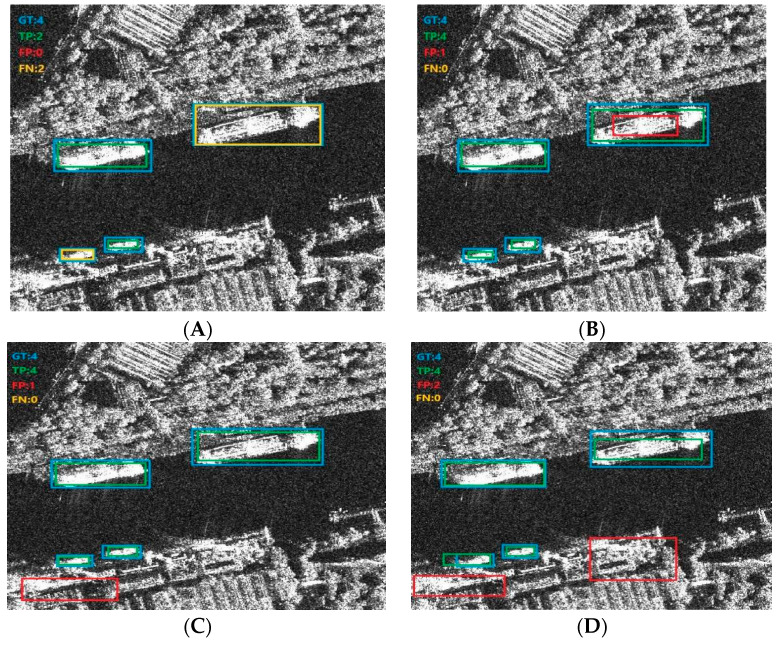

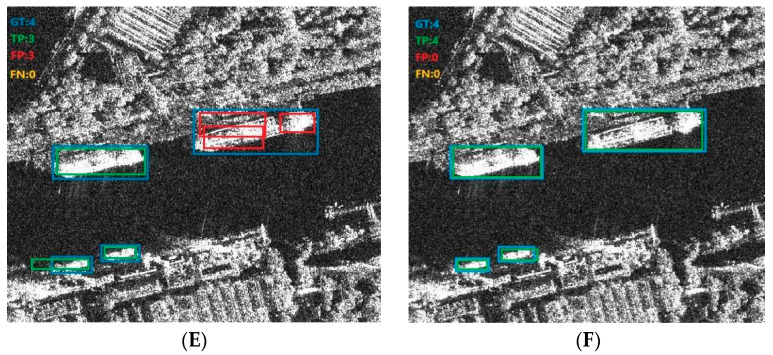
The detection conclusions of inshore ship. (**A**) Retinanet. (**B**) Dynamic R-CNN. (**C**) Faster R-CNN. (**D**) TOOD. (**E**) VFNet. (**F**) Our method.

To better demonstrate the advantages of the proposed approach, we compare the visualized conclusions of Retinanet, Faster R-CNN, Dynamic R-CNN, VFNet, and TOOD with our proposed approach, respectively. Figure 12 shows one example of detection conclusions of inshore ships which contain four ture ships. There are four TPs, zero FP, and zero FN in our method. Although the complex background of inshore ships brings difficulties to the detection, we can clearly see that our method identifies all correct targets without false alarms and missed targets, which well reflects the advantages of the proposed method.

**Figure 13:**
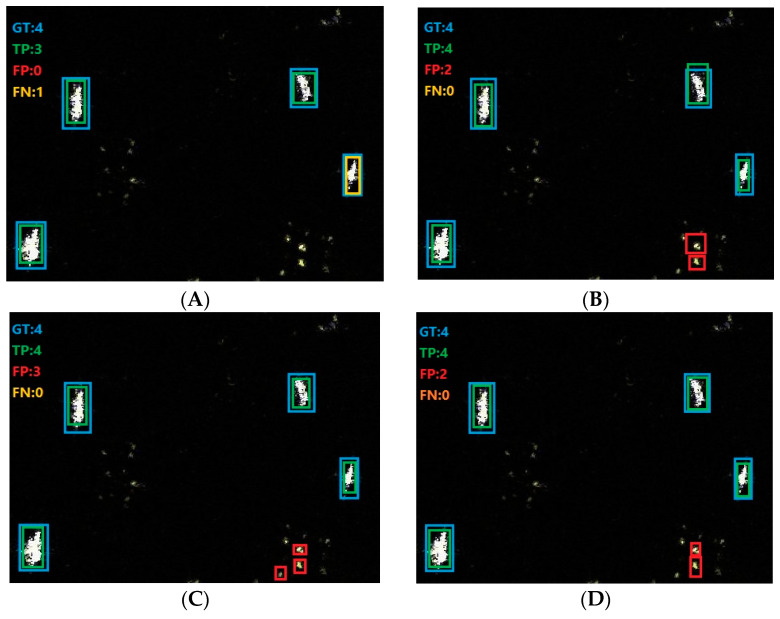

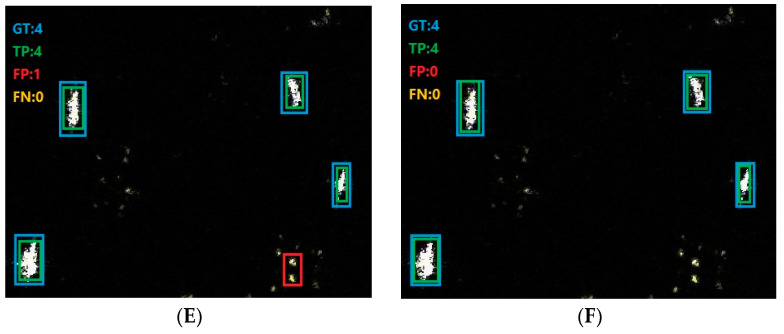
Detection conclusions of offshore ships. (**A**) Retinanet. (**B**) Dynamic R-CNN. (**C**) Faster R-CNN. (**D**) TOOD. (**E**) VFNet. (**F**) Our method.

Figure 13 shows one example of detection conclusions of offshore ships which contains four true ships. For offshore ship detection, some reefs are easily detected as false alarms, while our method identifies all correct targets without false alarms and missed targets, which reflects the advantages of our approach well.

**Figure 14:**
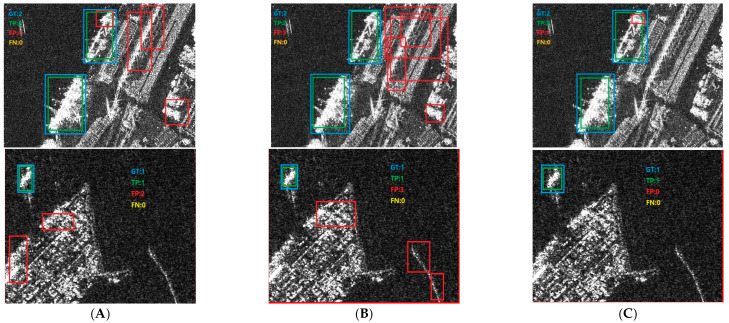
Detection conclusions of attention mechanisms. (**A**) SE. (**B**) CBAM. (**C**) MPCA.

To compare the effects of different attention mechanisms, we show the visualized conclusions with SE, CBAM, and MPCA in Figure 14, respectively. Here, in order to better demonstrate the effectiveness of MPCA, we have selected two examples. For SE as shown in (A), the number of FPs in the two images is 4 and 2, respectively. For CBAM as shown in (B), the number of FPs in the two images is 5 and 3, respectively, while for MPCA as shown in (C), the number of FPs in the two images is one and zero, respectively. Among these algorithms, MPCA has the fewest false alarms, which validates its effect. In addition, CBAM has the highest false alarm rate, which also validates our previous thinking that CBAM uses spatial attention and channel attention and channel attention can extract most of the ship’s features, but the use of spatial attention can reduce the correlation of ship features.

## 4. Discussion

SAR images have complex background noise, making it very difficult to improve detection accuracy. In the comparative experiment, we can clearly see that Faster R-CNN has an AP of 93.9%, Retinanet has an AP of 85%, Dynamic R-CNN has an AP of 93.7%, VFNet has an AP of 92.2%, and TOOD has an AP of 92.3%. Our work has improved the detection accuracy of SAR ship detection. Of course, this article still has some drawbacks, such as using the traditional RPN structure to generate candidate boxes, which lacks a certain degree of adaptability. In addition, in the ablation experiment, our method’s FPS is not optimal. Therefore, in the future, we will attempt to introduce adaptive candidate box generation to further improve detection accuracy and optimize algorithms to maximize FPS values.

## 5. Conclusions

In our paper, we use Convnext to extract superior multiscale feature maps. MPCA module is used to suppress false alarms. FIPN is employed for enhancing the semantic information of shallow feature maps. TLFI module is employed to make up for the lack of semantic information originating from channel diminution in the top-level feature map. Ablation experiments and comparative experiments demonstrate that our method has advantages over the previous methods. The AP of our method has reached 95.6%, and our method has increased AP by at least 1.7%.

## Figures and Tables

**Table 1:** Data of ablation experiments about Convnext and FIPN.

Backbone	Neck	FPS	AP (%)
Resnet50	FPN	**8.6**	93.2
Focalnet	FPN	5.6	91.9
Swin Transformer	FPN	6.5	93.9
Convnext	FPN	7.0	94.5
Convnext	PAFPN	**6.8**	92.5
Convnext	FPN_CARAFE	6.7	94.5
Convnext	FEFPN	6.3	93.2
Convnext	NASFPN	5.1	93.3
Convnext	FIPN	6.5	**95.4**

**Table 2:** Data of ablation experiments about TLFI.

TLFI	FPS	AP (%)
**×**	6.4	95.1
**√**	**6.5**	**95.4**

**Table 3:** Data of ablation experiments about MPCA.

Module	FPS	AP (%)
SE	**6.7**	95.3
CBAM	6.1	94.5
MPCA	6.2	**95.6**

**Table 4:** Data of comparative experiments.

Detectors	AP (%)
Faster R-CNN	93.9
Retinanet	85
Dynamic R-CNN	93.7
VFNet	92.2
TOOD	92.3
Ours	**95.6**

## Data Availability

Not applicable.
